# Microfluidic Applications in Drug Development: Fabrication of Drug Carriers and Drug Toxicity Screening

**DOI:** 10.3390/mi13020200

**Published:** 2022-01-27

**Authors:** Pei Zhao, Jianchun Wang, Chengmin Chen, Jianmei Wang, Guangxia Liu, Krishnaswamy Nandakumar, Yan Li, Liqiu Wang

**Affiliations:** 1 Energy Institute, Qilu University of Technology (Shandong Academy of Sciences), Jinan 250014, China; zhaop@sderi.cn (P.Z.); wangjc@sderi.cn (J.W.); chencm@sderi.cn (C.C.); wangjm@sderi.cn (J.W.); liugx@sderi.cn (G.L.); nandakumar@lsu.edu (K.N.); 2 School of Energy and Power Engineering, Qilu University of Technology (Shandong Academy of Sciences), Jinan 250014, China; 3 Cain Department of Chemical Engineering, Louisiana State University, Baton Rouge, LA 70803, USA; 4 Department of Mechanical Engineering, Faculty of Engineering, The University of Hong Kong, Hong Kong 999077, China

**Keywords:** microfluidic technology, drug development, drug carrier, micro/nanoparticles, drug toxicity screening, organs-on-chips

## Abstract

Microfluidic technology has been highly useful in nanovolume sample preparation, separation, synthesis, purification, detection and assay, which are advantageous in drug development. This review highlights the recent developments and trends in microfluidic applications in two areas of drug development. First, we focus on how microfluidics has been developed as a facile tool for the fabrication of drug carriers including microparticles and nanoparticles. Second, we discuss how microfluidic chips could be used as an independent platform or integrated with other technologies in drug toxicity screening. Challenges and future perspectives of microfluidic applications in drug development have also been provided considering the present technological limitations.

## 1. Introduction

With the development of science and technology, the pharmaceutical industry has entered the development mode of innovation-oriented drugs. However, the development of new and efficient drugs is a tedious process that generally consists of drug synthesis, drug delivery and drug evaluation [1]. Approximately USD 648.0 million and a duration of more than a decade are required to launch a new drug in the market [2]. Drug delivery aims to administer an optimum dosage of the drug in the body and achieve the expected therapeutic effects. Drug carriers play an important role in drug delivery. They deliver drugs to the target sites, ensure maximum efficacy of drugs and increase the duration of drug release. Drug evaluation mainly focuses on the toxicity, safety, pharmacokinetics and physicochemical properties of newly developed compounds [3]. Drug toxicity affects the safety of drugs in vivo, and toxicity screening is a key step in drug evaluation. Microfluidics can accurately control and manipulate sub-millimeter fluids, which has advantages in the preparation of micro/nanoparticles and the construction of extracellular microenvironments [4]. It has been progressively applied in the field of drug carrier development and drug toxicity screening.

A drug carrier is a drug-loaded material that can not only achieve high drug loading and encapsulation efficiency but also ensure controllable and targeted drug release, thereby reducing the toxic and side effects of drugs on the entire body [5,6]. Micro/nanoparticles are a group of new drug carriers that include micro/nanospheres and micro/nanocapsules and have shown prospects as a powerful and effective tool in drug delivery [7]. The size and morphology of the particles affect the spatiotemporal kinetics of drug release [8]. Conventional preparation methods such as mechanical stirring, spray drying and membrane emulsification rely on nonstandard multistep procedures that are time-consuming and expensive [9]. Microfluidic technology can exert precise control over the local particle-formation environment in a continuous flow pattern [10]. The mixing rate and the heat and mass transfer process of the fluid on a microfluidic chip can all be accurately controlled by designing the microchannel architectures, flow rate and viscosity at the interface between two phases [11,12]. Therefore, various micro/nanoparticles with unique properties could be synthesized using microfluidics [13]. In this review, the recent development pertaining to micro/nanoparticles used for drug delivery based on microfluidics is explained in detail.

In addition, numerous drug candidates require toxicity screening for determining their toxicity at the preclinical stage, which is essential for the safe development of novel drugs [14]. The traditional models used for toxicity screening consist of in vitro models (protein structure-based, cell-based) and in vivo models (animal tests and clinical trials) [15]. Animal models have drawbacks such as ethical issues, high cost and no scope of performing high-throughput assays. In addition, there are physiological and metabolic differences between animal models and humans [16]. Cell-based drug toxicity screening helps in eliminating and selecting from thousands of candidates before the use of in vivo models, thereby saving time and cost considerably [17]. Current methods used for cellular toxicity screening are based on Transwell assays and Dunn chambers, which have the disadvantages of simple cell culture conditions, time-consuming operations and the use of large volumes of reagents. Moreover, achieving complex drug combination screening and simulation of cell microenvironment is difficult [18,19]. Recently, microfluidic platforms were developed to address the challenges of cell-based drug screening, providing benefits such as reducing the volume of reagents required and developing a three-dimensional (3D) cell culture that is similar to the physiological/pathological microenvironment [1,20]. The application of microfluidic chips in drug toxicity screening is attracting increasing research attention [21]. Concentration gradient microfluidic chips could achieve spatiotemporal control of drug concentration, which plays an important role in drug toxicity screening. On the other hand, various types of cells have been integrated into microfluidic devices to produce organs-on-chip, which could reconstruct 3D tissue structures, blood flow and mechanical movement, as well as reproduce organ functions in vitro [22]. In this review, we have briefly explained the microfluidic platforms used in drug toxicity screening, including the drug dilution generator, 3D cell coculture and organs-on-chips.

**Figure 1:**
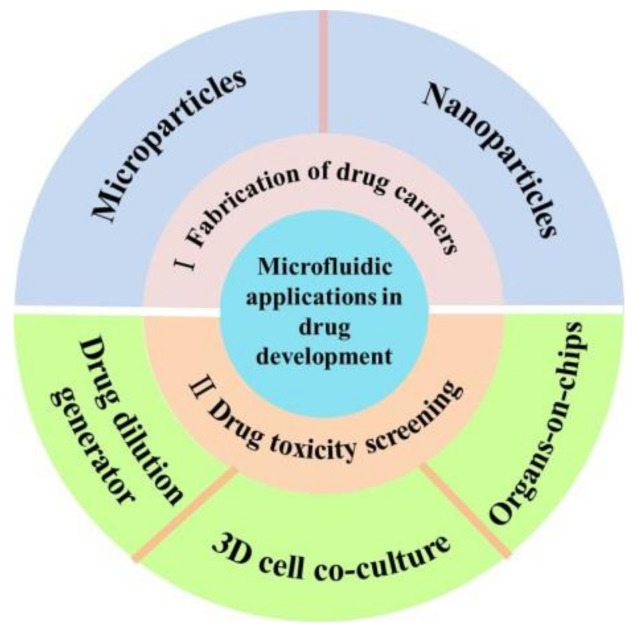
Schematic illustration of the contents of this review.

In this short review, we summarized the recent developments of microfluidic applications in drug development, including fabrication of drug carriers and drug toxicity screening (Figure 1). Finally, the major limitations and challenges in this area and opinions on its future developments have been proposed.

## 2. Microfluidics for the Fabrication of Drug Carriers

Drug carriers can deliver picoliter to nanoliter volumes of drugs, which is particularly important for improving the bioavailability of drugs and minimizing their adverse effects. Drug carriers can also ensure controlled or targeted drug release. In this section, the application of microfluidics for the development of drug carriers, including microparticles and nanoparticles, is explained in detail.

### 2.1. Microfluidics for the Fabrication of Microparticles

Microparticle size and morphology are crucial factors that affect drug encapsulation efficiency, drug release rate and in vivo biodistribution. Monodisperse microparticles with controllable morphology are the desired formulation in the medical and pharmaceutical fields [23,24]. However, the improvement in dispersity and the control over the morphology of microparticles by traditional methods (such as mechanical stirring and spray drying) is still challenging [25,26,27]. In the past two decades, microfluidics offered a low-cost and easy-to-use platform for controlling the fluid flow, and droplet microfluidics was extensively used to synthesize a large variety of microparticles [28,29]. In a microfluidic device, droplets were formatted by introducing a dispersed fluid into a continuous fluid. Viscous and inertial forces and flow rates are the main parameters of droplet generation. Microparticles are obtained by the solidification of droplets. The particle size can be adjusted within the range of tens to hundreds of micrometers, and the morphologies and micro/nanostructures of the particles can also be precisely regulated, including core-shell structure, porous structure, etc. [30]. Co-flow, cross-flow and flow-focusing are the basic passive droplet generators. Recent studies on the microfluidic preparation of microparticles are summarized in Table 1. In the preparation process of microparticles, microfluidic droplets serve as a soft template that bears physical or chemical crosslinking, enabling the synthesis of micro-scale materials with a flexible morphology and structure by using single/double/multiple emulsions [31]. In this section, the use of microfluidics for MP fabrication is discussed.

**Figure 2:**
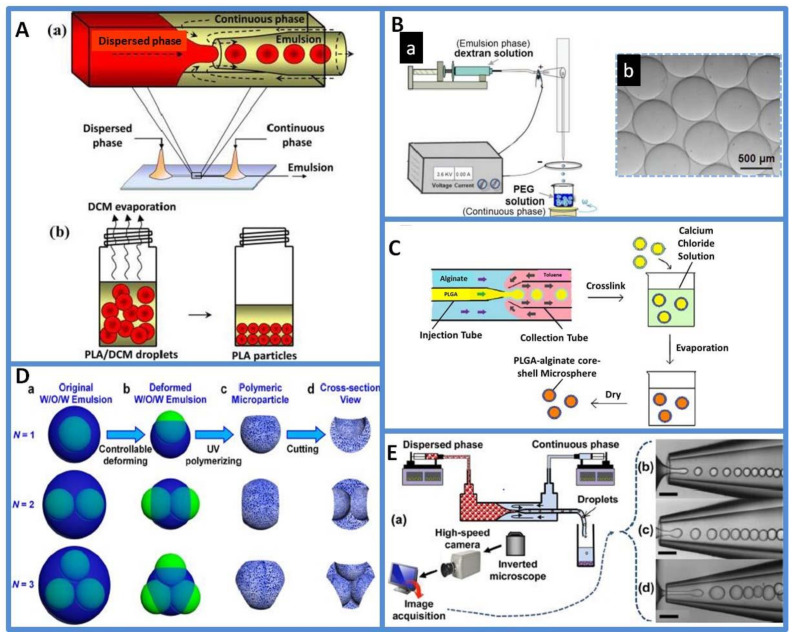
(**A**) Glass capillary microfluidics/solvent evaporation method performed to fabricate PLA particles. (**a**) The schematic diagram of the experimental setup with an expanded schematic of droplet formation in the flow-focusing region of the microfluidic device. (**b**) The formation of PLA particles from emulsion droplets via evaporation of dichloromethane (DCM) at room temperature. Reprinted from [33] with permission, Copyright 2015 American Chemical Society. (**B**) Generation of water-in-water (W/W) emulsions with tunable sizes via all-aqueous electrospray: (**a**) Schematic of the experimental setup. (**b**) Optical microscope images of the monodisperse W/W emulsions. Reprinted from [36] with permission, Copyright 2015 American Chemical Society. (**C**) Fabrication process of PLGA-alginate core–shell microspheres. Reprinted from [38] with permission, Copyright 2013 Elsevier. (**D**) Strategy for controllable fabrication of highly interconnected hierarchical porous microparticles. (**a**–**d**) The fabrication of microparticles with controllable micrometer-sized pore structures and shapes from controllably deformed W/O/W emulsions containing an oil phase that is partially miscible with the aqueous phases. The porosity and pore structure were separately tuned by changing the size and number (N) of the inner drops and the amount of the surfactant in the oil phase. Reprinted from [39] with permission, Copyright 2015 American Chemical Society. (**E**) (**a**) The experimental setup of a microfluidic device for the production and monitoring of microdroplet generation. (**b**,**c**) The monodispersed droplet generation in the dripping regime. (**d**) Polydispersed droplet generation in the jetting regime. Scale bars: 250 μm, Reprinted from [40] with permission, Copyright 2015 American Chemical Society.

Single emulsions are droplets of one phase fluid dispersed in another immiscible phase fluid. Various microfluidic devices have been developed to produce composite oil-in-water (O/W), water-in-oil (W/O) and water-in-water (W/W) single-emulsion templates [32]. In the flow-focusing devices, the disperse phase encounters the continuous phase at a crossing junction and monodisperse droplets are generated in the dripping state. Weitz et al. reported the production of polylactic acid (PLA) microspheres with controllable size-based flow-focusing glass capillary device by O/W emulsion, as shown in Figure 2A. The droplet size and generation rate were changed by controlling the phase flow rate and orifice size of the collection capillary. The obtained microspheres exhibited low coefficients of variation of particle sizes (CV < 5%) [33]. In another study, Ahmed et al. developed alginate hydrogel microspheres through W/O emulsion by using a PDMS-based microfluidic device. Alginate microspheres with sizes less than 30 μm were obtained. Alginate microspheres with a mean diameter in the range of 8 to 28 μm were successfully generated using microfluidic channels with various dimensions and by controlling the flow parameters [34]. As biocompatible formulations, W/W emulsions can better preserve the activity of biomolecules and viability of cells than the other two emulsions. W/W emulsions are formed when two incompatible solutes, such as dextran and polyethylene glycerol (PEG), are mixed [35]. Song et al. developed an all-aqueous electrospray approach to generate an aqueous two-phase emulsion droplet template to fabricate microparticles, as shown in Figure 2B. The diameters of the droplets decreased as the applied voltage increased or the diameter of the glass nozzle decreased. The size and structures of microparticles were adjusted by controlling the shape of the templates [36]. Overall, droplets and microparticles could be endowed with the desired size by adjusting the flow parameters and the design of the microdevice.

Microparticles with different morphologies and structures, including core-shell, porous and other complex morphologies, were generated by using monodisperse double- or multiple-emulsion droplets [37]. Double or multiple emulsions are droplets with smaller droplets encapsulated in large drops. Core-shell microparticles can be prepared by using double emulsions, such as W/O/W or O/W/O emulsions. A previous study reported the use of a microfluidic W/O/W emulsion to develop monodisperse rifampicin-loaded PLGA-alginate core-shell microspheres as delivery vehicles, as shown in Figure 2C. The size of the core and shells has a considerable impact on drug release kinetics. Moreover, core-shell microspheres create new opportunities to customize the release kinetics of active ingredients [38]. The W/O/W emulsions were also used for the template synthesis of porous microspheres with an interconnected hierarchical pore structure. Chu et al. developed a facile approach for the one-step fabrication of porous poly(methyl methacrylate-*co*-ethyl glycol dimethacrylate) microparticles, as shown in Figure 2D. The porosity and pore structure were separately tuned by changing the size and number (N) of the inner drops and the amount of the surfactant in the oil phase. The highly interconnected pores provide easy access for the protein molecules to diffuse and be released [39]. Another investigation reported the development of paracetamol-loaded multi-structured polymeric microparticles using glass capillary devices, as shown in Figure 2E. The rate of drug release was precisely controlled by the microstructure of the particles [40]. Compared with the traditional method for porous microsphere preparation, microfluidic emulsion could create controllable and customizable micrometer- and nanometer-sized pores that have large-scale application in drug delivery.

In general, droplet microfluidics is a powerful tool for the synthesis of microparticles with uniform size and customizable structure. These materials exhibit distinct functions and can be used in drug delivery. However, some limitations still remain; for example, the output is still too low to meet industrial production requirements. A series in-parallel connection was designed to maximize production efficiency. Joseph et al. realized a scalable and high-throughput generation of monodisperse microgels by using a parallelized step emulsification device [41]. Besides, the microfluidic device is another factor to consider for large-scale production. Although the preparation of a glass capillary is simple, its structural accuracy is low and affected by human factors, so it cannot be mass-produced. Compared with glass capillary devices, polydimethylsiloxane (PDMS) devices are more polytropic in their design for the material plasticity property, but they are not suitable for use with high-viscosity polymers and organic solvents.

### 2.2. Microfluidics for the Fabrication of Nanoparticles

In nanomedicine-based therapy, nanoparticles (NPs) are used as carriers for delivering drugs [45], siRNAs [9,46] or small molecular proteins [47] for treatment. The particle size, size distribution, shape, surface charge and components of nanoparticles have important effects on their colloidal stability, drug loading efficiency, release behaviors and cellular toxicity [48]. Conventional approaches for preparing NPs include nanoprecipitation, emulsification-based solvent evaporation and self-assembly of monomers. However, these methods are largely unstandardized, include multiple steps and are time-consuming. Nanoprecipitation is a common technique used for NP preparation. The NP-forming components are initially dissolved in a “good solvent” and after mixing with a “poor solvent”, precipitation is triggered and NPs are formed [49,50,51,52,53]. Ultrasound and homogenization are traditional nanoprecipitation methods, but these bulk preparation methods have the disadvantage of lack of control over experimental variables, and hence, the products have polydisperse distributions and large batch-to-batch variations [54]. Microfluidics offers a new approach for precisely controlling the preparation process of NPs and can be used to synthesize nanoparticles in a highly controlled and reproducible way [55]. Recent studies on the microfluidic preparation of NPs are summarized in Table 2 [10,56]. Diverse microfluidics methods have been developed for the preparation of NPs, such as flow focusing, chaotic flow, microvortices and droplet microfluidics. In this section, the use of microfluidics for the preparation of NPs is explained.

**Figure 3:**
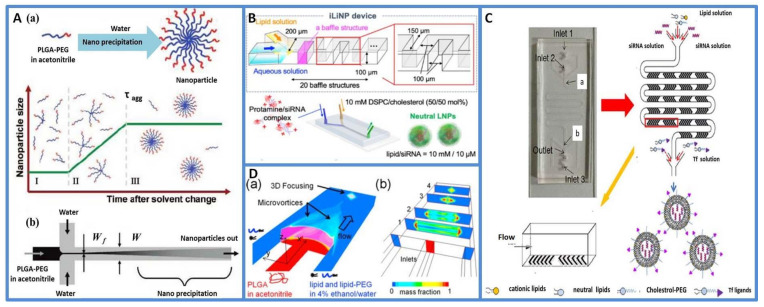
(**A**) Nanoprecipitation of PLGA-PEG copolymers. (**a**) Self-assembly process of PLGA-PEG nanoparticles. The process occurs in three stages: (**I**) nucleation of nanoparticles, (**II**) growth through aggregation and (**III**) development of kinetically locked nanoparticles after a characteristic aggregation time scale τ_agg_. (**b**) The process of mixing can be performed in a microfluidic device through hydrodynamic flow focusing. Reprinted from [51] with permission. Copyright 2008 American Chemical Society. (**B**) Schematic illustration of a microfluidic device for preparing siRNA-loaded noncationic NPs (called the iLiNP device) and the one-step production of the protamine/siRNA-complex-loaded neutral LNPs. Reprinted from [11] with permission. Copyright 2021 American Chemical Society. (**C**) Schematic of Tf-LNP synthesis using a 3-inlet microfluidic device. (**a**) The first Y-junction. (**b**) The second Y-junction, Reprinted from [46] with permission. Copyright 2017 Elsevier. (**D**) Mass production and size control of lipid–polymer hybrid (LPH) nanoparticles through controlled microvortices. (**a**) The schematic and (**b**) cross-section views of the microfluidic platform generated two symmetric microvortices. Reprinted from [62] with permission. Copyright 2012 American Chemical Society.

Hydrodynamic focusing is a powerful tool for mixing and diffusion-controlled chemical reactions, in which two miscible solvent fluids combine in a laminar flow pattern and then mix by diffusion at the interface. A common way to perform hydrodynamic focusing is to use three inlet microfluidics, where the core flow containing the samples of interest is sheathed by side fluids [57,58]. T- or Y-junction shapes are a common flow-focusing geometry. The microchannel architectures, flow rate and viscosity at the interface between two phases, polymer composition and concentration are the main parameters regulating the fluid-mixing process. Karnik et al. used T-junction microfluidic channels to control the nanoprecipitation process for the synthesis of drug-encapsulated biodegradable polymeric poly(lactic-*co*-glycolic acid)-polyethylene glycol (PLGA-PEG) nanoparticles, as shown in Figure 3A. The size of the nanoparticles formed by nanoprecipitation in bulk and by hydrodynamic flow focusing was compared. Considering that the polymer composition is the same, the size distribution of nanoparticles synthesized by bulk nanoprecipitation had a bimodal distribution with a broad size distribution (50 to 300 nm) in addition to 30 nm. On the other hand, the size distribution of the nanoparticles prepared by hydrodynamic flow focusing showed a unimodal distribution with the absence of larger aggregates [51]. Three-dimensional hydrodynamic flow focusing consisting of three sequential inlets for vertical focusing and a separate inlet for side sheath flow was also used to produce NPs. A previous investigation demonstrated that the 3D flow-focusing structure enabled the isolation of the precipitation polymer from the channel walls and successfully produced NPs even with the use of high-molecular-weight PLGA precursors [59]. Additionally, size-controlled lipid nanoparticle (LNP)-based DNA/RNA delivery was produced by the flow-focusing method. Kimura et al. reported a one-step method for the production of siRNA-loaded NPs, and the microfluidic device, called iLiNP, was able to encapsulate siRNAs into the noncationic NPs effectively and control the NP size precisely, as shown in Figure 3B. The results demonstrated that the LNPs did not cause cytotoxicity in vitro and the 40–50 nm sized NPs suppressed target protein expression at a dose of 20 nM of the siRNA [11]. In general, hydrodynamic flow-focusing systems are easy to develop and use. However, mixing efficiency and throughput scale limit the application of this method at the industrial level.

Some researchers have attempted to improve mixing efficiency and throughput by using chaotic flow and microvortices in microfluidic channels. In the chaotic flow method, geometric patterns are usually introduced to induce stretch and fold volumes of the fluid over the cross-section of a microchannel [60]. Typically, the staggered herringbone (SHM) structures use an array of “herringbone grooves” on one or more surfaces of a microchannel and can induce mixing within a continuous flow, thus favoring optimized product size and quality. Compared with hydrodynamic flow-focusing approaches at equivalent flow rate ratios, the SHM mixer exponentially increases the surface area between two fluids with distance traveled, resulting in faster diffusional mixing. Julian Thiele et al. developed an SHM mixer to develop NPs with polydispersity indexes as low as 0.02. They proved that the crystallinity and sizes of the product could be controlled by predesigning the collection distance of the spray [58]. Another study systematically studied a single-step microfluidic focusing process to synthesize LNPs. This process enabled the rapid and efficient synthesis of LNPs, as shown in Figure 3C. The products had a small size, uniform structure and high siRNA delivery efficiency in vitro and in vivo [46]. Moreover, the microvortex technique was developed to overcome the inefficiencies of slow diffusive mixing and realize large-scale nanoparticle production at high Reynolds numbers (>100) [61]. Kim et al. developed a pattern-tunable microvortex platform that allowed the mass production and size control of nanoparticles with superior reproducibility and homogeneity, as shown in Figure 3D. NPs could be produced up to 1000 times faster than conventional microfluidic diffusive syntheses [62].

In addition to the above methods, emerging acoustofluidic (the fusion of acoustic and microfluidics) methods were reported to synthesize NPs in a controllable and reproducible manner [63]. Huang et al. developed an acoustofluidic strategy for the synthesis of polymeric NPs, chitosan NPs, organic-inorganic hybrid nanomaterials, metal–organic framework biocomposites and lipid-DNA complexes. This method achieved active mixing of reagents by adjusting the strength of the acoustic streaming [64]. Moreover, some researchers created a 3D geometry microfluidic device to enhance the mixing effect and throughput scale. Nagasawa et al. designed a K-M mixer (K-M is the abbreviation of Kyoto University-MCPT (Micro-Chemical Process Technology Union)). In the K-M mixer, reactant fluids are divided into fluid segments by relatively small channels to reduce diffusion length, and the fluid segments then collide at a single point so that shear is applied to them. It feathered rapid mixing, high throughput and a broad range of mixing ratios and is clogging-proof [65]. Anton et al. used micromixer in the preparation of polymethyl methacrylate nanoparticles. The NPs had a particle size of about 100 nm and could achieve encapsulate high levels of a lipophilic drug (ketoprofen) [49].

In general, microfluidics provides a means to implement controllable and reproducible fabrication methods for nanoparticle production, and the obtained NPs shows superior performance in drug delivery system. However, many challenges still remain, such as the production rate and throughput. Jong-Min et al. designed a coaxial turbulent jet mixer in the synthesis of homogeneous-sized NPs at high production throughput up to 3 kg/day [66]. On the other hand, PDMS-based flow-focusing platforms are unsuitable for organic solvents, limiting the fabrication of some polymeric nanoparticles.

### 2.3. Outlook and Challenges

To summarize, microfluidics is a novel method for the controllable and reproducible fabrication of micro/nanoparticles, and the products can be used as drug carriers. Firstly, the process usually includes ultrasmall sample volumes of reagents, greatly reducing research and development costs. The products can deliver drugs at nanoliter volumes, which is particularly important for improving the bioavailability of the drugs and minimizing their adverse effects. Secondly, the products are homogenized micro/nanodroplets, which is significant for pharmacokinetic consistency. On the other hand, multiple and structured drug-loading MPs for therapeutic purposes could be designed and developed.

Nevertheless, the transformation of microfluidic micro/nanoparticles from an academic research level to the industrial and clinical levels still faces great challenges. First, the production rate and products require consideration. Conventional fabrication methods have the relative advantage of easy scalability, in contrast. On the other hand, microfluidics-based methods can achieve precise control of particle size and structure at the cost of yield volumes. Current attempts at incorporating high-throughput production within a microfluidic platform have been undertaken by parallelization, but challenges pertaining to reproducibility and output quality still remain. Moreover, the biocompatibility and toxicity analysis of micro/nanoparticles is also essential. Material selection and solvent removal for the process of preparation are the top priority. With the development of material science and microfluidic technology in the future, we expect dramatic development in microfluidic micro/nanoparticle drug delivery systems.

## 3. Microfluidics for Cell-Based Drug Toxicity Screening

Apart from drug carriers, drug toxicity screening is of immense interest and importance in the drug development process. In this section, microfluidic platforms for drug toxicity analysis are introduced, including a drug dilution generator, 3D cell coculture and organs-on-chips.

### 3.1. Drug Dilution Generator Based on Continuous-Flow Based Microfluidics

In an in vivo environment, the concentration gradient of biomolecules regulates cell behaviors such as cell growth and cell differentiation. Therefore, drug toxicity screening usually requires the study of dose-dependent cellular responses at different drug concentrations. Traditional experiments on drug concentration gradient are primarily performed in multiwell plates by manual dilution, which is expensive and time-consuming [75]. Due to the development of microfluidics, concentration-gradient-based microfluidic chips can achieve spatial and temporal control of drug concentration. They increase throughput, reduce experimental costs and possess a higher gradient resolution [76].

**Figure 4:**
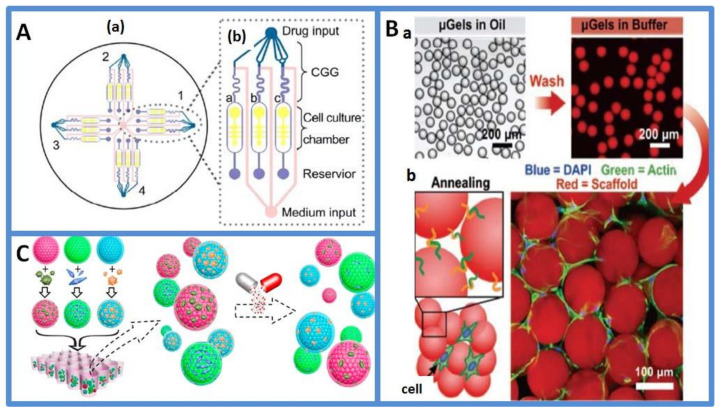
(**A**) Schematic of the integrated microfluidic device for a drug sensitivity test. (**a**) The device consists of four uniform-structure units (1,2,3,4) connected by a common reservoir in the center of the device. (**b**) Magnified section of one structural unit containing an upstream concentration gradient generator and downstream parallel cell culture chambers, a,b,c are different concentrations of the drug. Reprinted from [80] with permission, Copyright 2013 Elsevier. (**B**) An overview of monodisperse microgel (μgel) production and in situ scaffold formation. (**a**) Crosslinked μgels separated from the oil phase and washed. (**b**) μgels seeded with cells in the solution and covalently linked together in situ to form cell-laden microporous scaffolds. Reprinted from [41] with permission, Copyright 2019 Wiley. (**C**) Schematic diagram of the cell spheroids-on-barcodes platform for drug screening. The GelMA hydrogel-encapsulated green, red and blue PhC barcode particles were first cultured with HCT-116, NIH-3T3 and HepG2, respectively. Reprinted from [82] with permission, Copyright 2016 American Chemical Society.

Based on continuous-flow microfluidics, researchers have designed various delicate microfluidic chips to achieve precise and controlled drug distribution for drug toxicity screening [77]. In 2004, Hung et al. first developed a microfluidic cell culture array that integrated a concentration gradient generator for long-term cellular-activity monitoring [78]. Although this microfluidic cell culture array was not used in drug testing, the ability of the long-term cell culture was applied in drug screening. In another study, a serial dilution microfluidic chip was constructed to generate linear or logarithmic stepwise drug concentrations for drug toxicity assays. The microfluidic chips were operated using a fluorescence microscope, and the linearity of linear or logarithmic dilution was analyzed by fluorescence measurements. The results of the cytotoxicity test of breast cancer drugs using the logarithmic dilution chip were consistent with the results of the manual dilution experiment [79]. Concentration gradient generators were also integrated with cell co-cultured chips for drug toxicity analysis. Xu et al. designed a microfluidic platform for drug sensitivity tests, as shown in Figure 4A. Lung cancer cells and stromal cells were co-cultured in a concentration gradient generator to assay anticancer drug efficacy under continuous supplementation of medium [80]. However, 2D concentration gradient microfluidics chips have limitations in the analysis of the toxicity of multidrug combinations. A previous study reported microfluidic chips with a helical structure. The chips achieved rapid mixing of solution, and the effect of four-drug combinations of 36 different concentrations on the viability of cells was tested on chips [81].

To conclude, compared with traditional multi-well plate assays, the concentration-gradient-based microfluidic chip has the following advantages in drug toxicity analysis: First, the concentration range is wide, and the gradient environment is relatively stable. Second, the concentration gradient can change dynamically with time, and the cell state can be monitored in real time to meet different needs. However, the system lacks the microenvironment of cell growth and differentiation, which may largely deviate from the therapeutic effect of the resulting drugs when applied to clinical trials.

### 3.2. Three-Dimensional Cell Coculture on Droplet-Based Microfluidics

A multi-well plate for drug toxicity screening refers to cells cultured in monolayer conditions, which have different behaviors than those observed in in vivo conditions. Cells are subject to the properties of a surrounding extracellular matrix, including biochemical and mechanical stimuli [83]. Recent trends show that several pharmaceutical companies are using 3D co-culture technologies to construct a mimicked microenvironment for drug toxicity analysis, such as cell encapsulation in hydrogel or microspheres.

Droplet-based microfluidic systems focus on generating and manipulating discrete droplets. Drug screening using droplet microfluidics is based on separate manipulation models of cell encapsulation [84,85,86], droplet merging [87,88], droplet mixing [89,90], on-chip incubation [91], droplet sorting [92] and multiple compound introduction [93]. Droplet-based microfluidic systems allow the assay to be multiplexed and increase the throughput of drug screening. Therefore, realistic drug testing (for example, a compound library against one specific cell line) can be performed on time to pick up the rare hit. Brouzes et al. constructed a representative integrated droplet-based workflow for high-throughput screening and on-chip cell viability assay [94]. They studied cells within intact droplets using a standard fluorescent assay, which was performed by laser line illumination and detection with photomultiplier tubes. A large number of cell droplets could be formed in a short time on microfluidic chips, thus improving experimental flux and effectively reducing reagent consumption. Nevertheless, for both cell culture and drug stimulation was completed in the microfluidic droplets, nutrient renewal and cell metabolite cleaning were the possible challenges in the droplet. Dino Di Carlo et al. presented the scalable high-throughput production of bioactive microgels for the formation of microporous tissue scaffolds in situ, and it is shown in Figure 4B [41]. These microporous structures enhanced nutrient and waste transport and led to immediate cell infiltration. Moreover, the types of cell culture (2D and 3D) had considerable effects on the results of drug stimulation. Patra et al. pretreated the device surfaces resistant to cell adhesion using oxygen plasma and a hydrophobic agent. Hence, human hepatocellular carcinoma cells could not stick to the wall and form tumor spheroids in the chamber. The effects of three anticancer drugs on tumor spheroids were studied [95]. Later on, researchers loaded cells into microspheres for drug toxicity analysis. Dhamecha et al. cocultured A549 lung adenocarcinoma cells and MRC-5 human lung fibroblasts on porous polymeric microspheres. In addition, a series of anticancer drugs were tested in an in vitro lung cancer model and 2D co-culture system. The chemoresistance of the tested anticancer drugs in the in vitro lung cancer model was greater than that in a monolayer coculture [96]. Yet, most of these co-culture system are based on a single organ cell source, so they are incapable of simulating organ-organ communication and unable to predict the effect of drugs on non-target tissues. Fu et al. presented a novel methacrylated gelatin hydrogel encapsulated core-shell photonic crystal (PhC) barcode particle with cells from different organ sources, as shown in Figure 4C [82]. The PhC cores of the barcode particles provided a stable diffraction peak that encodes different 3D cell aggregation types and distinguishes the biological responses of these cells during drug testing. The cytotoxic effect of two drugs was studied on barcode particles by fluorescent microscope and MTT assay. The results indicated that the cell spheroids-on-barcodes platform was quite promising for drug development.

The advantages of droplet-based drug screening assay include low reagent consumption, high throughput and controlled cell concentration. Compared with 2D traditional cell culture models, the 3D cell coculture models provide a microenvironment that more closely resembles in vivo conditions, such as cell-cell communications and cell-matrix interactions. These systems increase in vitro drug screening accuracy [97,98]. However, a big gap exists between the 3D cell screening model and the actual microenvironment of cells in the body. To overcome this gap, organs-on-chips with different structures were constructed to further simulate the functions of human organs.

### 3.3. Organs-on-Chips

Organs-on-chips are microfluidic cell culture devices capable of simulating the activities and physiological responses of an entire organ [21,99]. They can not only mimic the structure of real organs but also realize the physiological/mechanical dynamics of the human body [100,101]. Organs-on-chips use human-originated cells to minimize cross-species differences between the animal model and human organs [17].

Researchers have proposed methods for constructing organ-on-chip systems for mimicking their counterparts in vivo, including the brain [102], heart [103], lung [104,105,106], liver [107], kidney [108] and blood vessel [109]. Organs-on-chips used in drug development are summarized in Table 3. The liver is an important organ for drug metabolism and is highly susceptible to injury from drugs. Hepatotoxicity is also an important aspect of people’s interest in using organs-on-chips [110]. The liver-on-a-chip provided a scale-down strategy for recreating tissue microenvironment, representing an exciting potent platform for studying in vitro drug hepatotoxicity. Lee et al. developed an artificial liver sinusoid with a microfluidic endothelial-like barrier and achieved physical separation of the cell culture and nutrient transport compartments. Studies pertaining to diclofenac toxicity on chips showed no hepatotoxicity. Compared with the current microfluidic liver sinusoid, this chip created a mass transport environment and improved hepatocyte viability [111]. In another study, a 3D hepatocyte chip based on multiplexed microfluidic channels was developed. A concentration gradient generator was incorporated in the chip, which enabled the in vitro dose-dependent drug responses to predict in vivo hepatotoxicity. The reagent concentration in each channel was determined by measuring the steady-state fluorescence intensity of a tracer dye, and the viability of hepatocytes was quantitatively determined by a dual-nuclear staining method [108]. Generally, the liver-on-a-chip created a mass transport environment and improved hepatocyte viability compared with well-plate assays. The chip provided a new opportunity for understanding drug toxicity at the cellular and molecular levels in vitro.

**Figure 5:**
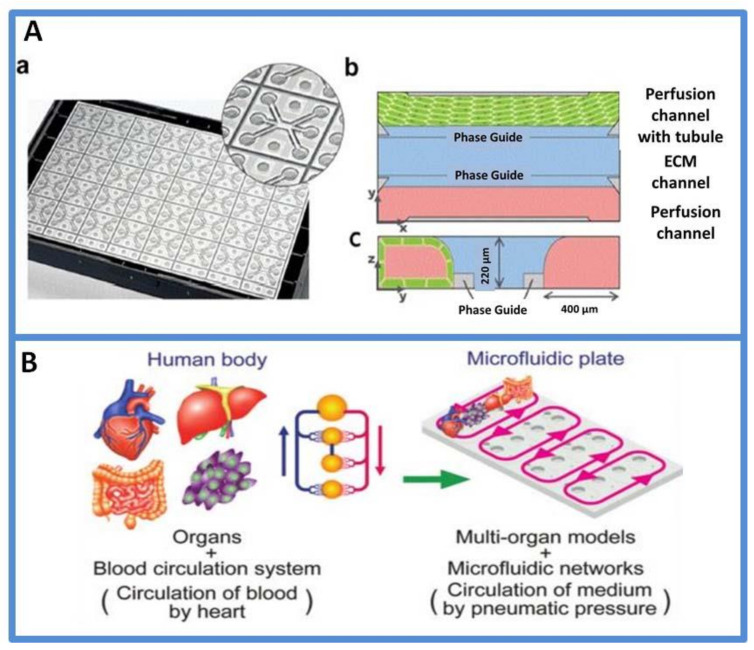
(**A**) Schematic depiction of the used microfluidic device (called OrganoPlate). (**a**) Image of the back side of the 3-lane OrganoPlate. The microfluid network is positioned between a glass sandwich of two microscope-grade glass plates attached to the bottom of a standard 384-titer well plate. Access to the microfluidic system is facilitated via the top wells. One OrganoPlate comprises a total of 40 chips. (**b**) Schematic of one chip presenting two perfusion channels with an extracellular matrix (ECM) channel in the middle. (**c**) The top perfusion channel represents the apical side of the epithelial barrier. Reprinted from [114] with permission, Copyright 2021 Elsevier. (**B**): A multithroughput multiorgan-on-a-plate system. Reprinted from [120] with permission, Copyright 2017 Royal Society of Chemistry.

In preclinical development, 2% of all candidate drugs fail because of nephrotoxicity [112]. Therefore, clinically predictive models for nephrotoxicity are required for early drug development. Nephrotoxicity of some drugs in vitro was evaluated using a kidney-on-a-chip using human proximal tubular cells. Vormann et al. assessed a 3D microfluidic platform for the detection of drug-induced kidney injury, as shown in Figure 5A. Four model nephrotoxic drugs (cisplatin, tenofovir, tobramycin and cyclosporin A) were tested in the chip. Measured parameters included cell viability, release of lactate dehydrogenase and N-acetyl-*b*-D-glucosaminidase, barrier integrity, release of specific miRNAs and gene expression of toxicity markers [113,114]. In another study, a kidney-on-a-chip was used to investigate the pathophysiology of drug-induced acute kidney injury and provide an effective assessment of drug-absorption-related nephrotoxicity. Therefore, the kidney-on-a-chip is a valuable tool for replacing animal testing. Besides, micro-engineered vascular systems are also important for the efficient assessment of the response of drug candidates to physiological barriers lining micro-vessels [115]. In the design of vascular chips, mechanobiology is an important factor to be considered [116]. For example, shear stress is the important hemodynamic force encountered by endothelial cells in a vessel and influences the functional behavior of vascular cells [117]. Gupta et al. designed the patterned silk films with microgrooves to induce the unidirectional alignment of vascular cells, mimicking their native form [118]. Zhang et al. created stable biodegradable scaffolds with embedded branching microchannel networks (called AngioChips) [119]. Endothelial cells were cultured in AngioChips to establish a stable and permeable vascular network. The spatial structure of the vascular chip was fine-tuned to mimic the anisotropy of native tissues, such as cardiac and hepatic tissues.

Collectively, organs-on-chips are a promising technique for drug discovery and development, especially in the prioritization of lead candidates and toxicity testing. Single-organs-on-chips provide valuable information in analyzing drug effects. However, the human body as a whole includes multiple organs and involves “cross-talks” among different tissues/organs. For systematically featuring drug responses on multiple tissues at one time, organs-on-chips are evolving toward multi-organs-on-a-chip [121,122,123]. Satoh et al. reported a pneumatic-pressure-driven platform for multi-throughput multi-organ experiments, as shown in Figure 5B. An eight-throughput two-organ system and a four-throughput four-organ system were constructed based on a common platform using different microfluidic plates [120]. Overall, the development of organs-on-chips for drug toxicity assays creates a potent strategy for understanding the fundamental knowledge of in vivo models.

### 3.4. Outlook and Challenges

Microfluidics has many advantages in drug dilution generators, 3D cell coculture and organs-on-chips and is a potent platform in the pharmaceutical industry. However, it has some limitations and challenges before conversion to product. First, the sources of cells to be introduced into cell culture should be considered. Cell lines cultured in a microfluidic device are usually derived from cancer cells and have markedly lost their original organ functionality. Primary human-derived cells have been used for predicting highly accurate pharmacokinetics but are limited because of donors and cost. Human-derived pluripotent stem cells offer an option for the problems such as the loss of functionality and moving organs-on-chips close to practical application. Second, materials used for device fabrication are another consideration. PDMS is a commonly used material but is limited by the absorption of drugs in drug assays. Commercially attractive materials such as injection-molded polymers or thermoplastics may solve this problem. Finally, the common detection schemes for microfluidics-based drug screening evaluation are usually off-line optical (such as microscopy) and electrochemical (such as impedimetric detection) and are possibly coupled to a mass spectrometric readout. However, a practical in vitro model should be a system with the real-time recording of various physiological responses to specific biological stimuli. Next, organs-on-chips should be integrated with on-chip sensors and actuators.

## 4. Conclusions and Future Perspectives

Microfluidic technology has been evolving rapidly over the past decade. Microfluidics can provide precise control over non-dispersive flow dynamics with high stability, which is quite useful for the synthesis of various micro/nanoparticles with unique properties. Meanwhile, the compartmentalization, high-throughput characteristics and construction of cell microenvironment have enabled its applications in early drug cytotoxicity evaluation and pharmacokinetics detection. Overall, microfluidics has developed as a powerful and promising platform for accomplishing two important aspects of drug development, namely in the generation of new drug entities as well as in drug toxicity screening in vitro.

Despite the spectacular achievements, challenges remain in this field. Most of the studies are still in the stage of laboratory research and have not reached the level of commercialization and generalization. Much more effort is required in developing, improving and promoting this technique in drug development. First, the regulatory bodies (FDA, EMA, etc.) are an important consideration. Lack of regulatory tools makes it difficult to evaluate common risks associated with microfluidics-based approaches. Therefore, considerable efforts are required for fostering a consistent microfluidics-based assessment. Second, the high-throughput and scalable production of micro/nanoparticles is still a problem that needs to be solved. Although parallelization realized high-throughput production, reproducibility and output quality are still problems. Third, seamless integration of separate modules into one systematic device is required, as this development would make microfluidics a more convenient, reliable platform for drug development. Fourth, organs-on-chips are integrated with on-chip sensors and drivers to develop the next-generation cell-based on-chip drug detection platforms. Considering the distinctive properties of this technique and considerable demand in applications, microfluidics may fundamentally modify the way of future micro/nanomanufacturing and drug screening in drug development.

## Figures and Tables

**Table 1:** Representative examples of microparticles fabricated using microfluidics.

Microfluidic Channels	MPs Synthesized	Diameter	Flow Rate (*F*)/Ratio	Reference
Flow focusing	Alginate microgels	~35 μm	*F*_aqueous phase_ = 20 μL/h *F*_oil phase_ = 150 μL/h	[42]
T-junction	Silica microspheres	~90–108 μm	*F*_continuous phase_=5 mL/h, *F*_dispersed phase_ =0.5 mL/h	[43]
Cross-junction	Alginate microspheres	150 μm	*F*_aqueous phase_ = 30 μL/h *F*_oil phase_ = 500 μL/h	[42]
T-junction	Alginate microspheres coencapsulated with superparamagnetic iron oxide NPs and dual drugs	~500 μm	——	[44]
Counter-current flow focusing	PLA and PLGA microparticles	4–30 μm	*F*_oil phase_ = 0.02–1.5 mL/h *F*_aqueous phase_ = 0.25–8 mL/h	[40]
Parallelized step emulsification device	Microgels	50 and 90 μm	*F*_total_ = 30 mL/h	[41]
Flow focusing	PLGA-alginate core–shell microspheres	15–50 μm	*F*_polymer phase_ = 0.8 mL/h *F*_aqueous phase_ = 2–8 mL/h	[38]

**Table 2:** Representative examples of nanoparticles fabricated using microfluidics.

Fabrication Method	NPs Synthesized	Diameter of NPs	Flow Rate (*F*)/Ratio	Reference
K-M impact jet mixer	Superparamagnetic iron oxide nanoparticles-loaded PMMA NPs	~100 nm	*F*_polymer_ = 0.2 mL/min *F*_water_ = 2–4 mL/min	[12]
3D hydrodynamic flow focusing	PLGA-PEG NPs	30–230 nm	*F*_organic_:*F*_water_ = 1:10	[67]
Flow focusing	Alginate nanogels	68–138 nm	*F*_organic_:*F*_water_ = 0.02–0.2	[68]
Parallel flow focusing	MPEG-PLGA NPs	50–200 nm	*F*_water_ = 5.0 mL/h *F*_polymer_ = 0.5–2.0 mL/h	[69]
Microfluidic flow focusing	PLGA-NPs	90–160 nm	*F*_diserse_/*F*_continuous_ = 50:5000–10,000	[45]
Tube-in-tube microchannel reactor	Amorphous cefuroxime axetil NPs	~440–760 nm	*F_tota_*_l_ = 1.5–6 L/min	[70]
Flash nanoprecipitation (FNP) mixing	Polystyrene NPs	Sub-150 nm	*F*_polystyrene_ = 2mL/s *F*_water_ = 2mL/s	[6]
Staggered herringbone mixer structures (SHM)	siRNA LNPs	~70–80 nm	*F*_ethanol_ = 0.5 mL/min *F*_water_ = 0.5–4.5 mL/min	[71]
Staggered herringbone micromixer (SHM)	Liposome	~50–450 nm	*F*_total_ = 0.5–6 mL/min	[72]
Fluidic nanoprecipitation system (FNPS)	Polymeric Janus NPs	Sub-μm	*F*_polymer_ = 100 μL/h *F*_water_ = 75 mL/min.	[5]
Coaxial turbulent jet mixer	PLGA-PEG NPs, lipid vesicles, iron oxide NPs, polystyrene NPs and siRNA/PEI polyplex NPs	≤100 nm	*F*_total_ = 300–500 mL/min	[66]
Millisecond microfluidic mixing	LNP-siRNA	≤100 nm	*F*_ethanol_ = 0.5 mL/min *F*_aqueous_ = 1.5 mL/min	[10]
Swirling microvortex reactors	LPNPs	~50 nm	*F*_total_ = 15 μL/min	[61]
Gas/liquid Taylor flow micromixer	LNP	~70 nm	*F*_total_ = 100–700 μL/min	[10]
Cross-junction T-junction	Itraconazole NPs	130–340 nm	*F*_continuous_ = 10–250 μL/min *F*_disperse_ = 50 μL/min	[73]
Microfluidic mixing	pH-sensitive LNPs	30 nm 100 nm 200 nm	*F*_lipid_ = 0.375 mL/min *F*_acetate_ = 1.125 mL/min	[74]

**Table 3:** Overview of organs-on-chips in drug testing.

Organ	Model	Chips	Drug Tested	Benefits	Reference
Brain	Blood–brain barrier	OrganoPlate	Organophosphate	High throughput and high-content imaging	[102,124]
Heart	Cardiac microphysiological systems	PDMS chips	Cardiac drugs	High throughput, simplify drug tests	[103]
Lung	Pulmonary edema-on-a-chip	PDMS chips	Angiopoietin-1 and transient receptor potential vanilloid 4	Reproduce the intra-alveolar fluid accumulation, fibrin deposition and impaired gas exchange	[105]
Liver	Liver sinusoid with a microfluidic endothelial-like barrier	Chips including glass bottom, silicone middle and acrylic top	Diclofenac	Create a mass transport environment, improve hepatocyte viability	[111]
Kidney	Kidney proximal tubule-on-a-chip	PDMS chips	Cisplatin	Enhance epithelial cell polarization and primary cilia formation	[125]
Vascular vessel	Scaffold with a built-in branching microchannel network (AngioChip)	Poly(octamethylene maleate (anhydride) citrate) chips	Terfenadine	Tunable elasticity and permeability, enable extensive remodel	[115,119]
